# Protocols for the obvious: Where does it start, and stop?

**DOI:** 10.1186/s13613-017-0264-7

**Published:** 2017-04-14

**Authors:** Armand R. J. Girbes, Paul E. Marik

**Affiliations:** 1 Department of Intensive Care, VU University Medical Center, PO Box 7057, 1007 MB Amsterdam, The Netherlands; 2 Division of Pulmonary and Critical Care Medicine, Eastern Virginia Medical School, Norfolk, VA 23507 USA

**Keywords:** Protocols, Quality improvement, Intensive care, Safety

## Abstract

Protocols can be helpful in specific situations and may have show benefits in clinical trials. So-called evidence based protocols and checklists frequently remind clinicians to do the obvious, but may also contain as part of a bundle, elements that are not based on the best current evidence. However, so called quality improvement programs frequently call for implementation of the total bundle. We think this is basically wrong and warn against that practice.

Improving the care and desired outcome of the intensive care patient is a continuous task for the team of physicians and nurses in the ICU. Skilled and dedicated professionals, continuous education and open communication between all workers in the ICU are essential for the best outcomes of critically ill and injured patients. Prevention and correction of errors and the recognition of systems errors with open reporting and process improvement all help to establish this task. However, it is essential to realize that in the end this all happens at the bedside of the patient. Protocols and checklists can be helpful at the bedside, for repeated simple tasks and as a support for inexperienced healthcare providers. Protocols should be in our view a way to support the implementation of the best up-to-date knowledge for a consistent treatment of patients. The call for “evidence-based protocols” for almost every clinical intervention is pervasive and never ending, being promoted by medical professionals but also managers, administrators, healthcare insurance companies and the “Quality Movement”. If a specific published protocol demonstrates an improvement of outcome, it is likely that the protocol will become mandated into daily clinical practice by various “stake holders” and regulatory agencies. However, when examining many of these studies, it is noteworthy that in many instances the baseline practice was very poor and that simple measures such as hand washing, using maximal barrier precautions when inserting a central line or making daily rounds in the ICU was all that was required to improve the outcome measure [1]. Protocols and checklist that remind clinicians “to do the obvious thing” will be of benefit for the patient. However, these simple measures are often “bundled” with other interventions that might be useless or even dangerous, but since the total outcome showed improvement, the “evidence-based” protocol is considered to improve outcome and may then be enforced on a national or international level; this may prove harmful particularly in high-functioning ICUs. Furthermore, quality and regulatory bodies frequently require compliance with all elements of the “bundle,” even those that may be potentially harmful. These organizations maintain that if all the elements of the “bundle” are not met no credit should be given for any of the elements. There are, however, no scientific data to support this concept. Donald Berwick’s assertion that “the movement to all-or-none performance assessment is an important milestone in the journey to high quality health care,” is potentially dangerous and may not improve patient outcomes when simple common-sense interventions are packaged with other more complex interventions that are unproven or harmful [2]. We have previously underscored the risk of regression to the mean with such protocols, meaning that in well-performing ICUs the introduction of so-called evidence-based protocols will compromise care and impede progress and innovation [3].

In their search to improve care in their ICU’s, Bodi et al. [4] studied the concept of having a senior physician tasked on a regular basis with ensuring that patients received appropriate care using a checklist of 37 safety measures. In this study, the only measurable improvement of outcome was a reduction in ventilator-associated pneumonias (VAP). It should be noted that the diagnosis of VAP is subjective and notoriously unreliable. Furthermore, the frequency of VAP at the start of the study was rather high and this reduction in VAP did not translate into a reduction in ICU length of stay (LOS) or any other outcome variable. In addition, one can argue that all critically ill patients deserve to be managed by well-trained and competent intensivists who do not need checklists to remind them to prescribe the appropriate medications in the correct dosages and to assess their patients renal function, fluid balance, hemodynamic status and level of sedation and pain.

We suggest that the take-home message of this study is that one should always strive to improve patient outcomes and that extra attention of nurses and intensivists at the patient’s bedside together with good communication will lead to better outcomes. The authors should be congratulated for their effort, but we caution against the adoption of this “protocol” in other units. Furthermore, this study highlights the fact that protocols and checklists frequently contain elements that are not based on the best current evidence. Gastrointestinal prophylaxis is an outmoded concept that should be abandoned. Likewise the role of tight glycemic control in the critically ill is of dubious benefit. Oral hygiene with chlorhexidine appears to be of limited clinical benefit and may increase the mortality of non-cardiac surgery patients. There is little data that targeting specific national goals improves patient outcomes, with parenteral nutritional having a vanishingly small role in the management of critically ill and injured patients [5].

## Abbreviations

ICU: intensive care unit
LOS: length of stay
VAP: ventilator associated pneumonia
